# Deep learning and explainable AI for classification of potato leaf diseases

**DOI:** 10.3389/frai.2024.1449329

**Published:** 2025-02-03

**Authors:** Sarah M. Alhammad, Doaa Sami Khafaga, Walaa M. El-hady, Farid M. Samy, Khalid M. Hosny

**Affiliations:** ^1^Department of Computer Sciences, College of Computer and Information Sciences, Princess Nourah bint Abdulrahman University, Riyadh, Saudi Arabia; ^2^Department of Information Technology, Faculty of Computers and Informatics, Zagazig University, Zagazig, Egypt; ^3^Department of Horti Culture, Faculty of Agriculture, Zagazig University, Zagazig, Egypt

**Keywords:** deep learning, explainable AI, grad-CAM, potato leaf disease classification, transfer learning

## Abstract

The accurate classification of potato leaf diseases plays a pivotal role in ensuring the health and productivity of crops. This study presents a unified approach for addressing this challenge by leveraging the power of Explainable AI (XAI) and transfer learning within a deep Learning framework. In this research, we propose a transfer learning-based deep learning model that is tailored for potato leaf disease classification. Transfer learning enables the model to benefit from pre-trained neural network architectures and weights, enhancing its ability to learn meaningful representations from limited labeled data. Additionally, Explainable AI techniques are integrated into the model to provide interpretable insights into its decision-making process, contributing to its transparency and usability. We used a publicly available potato leaf disease dataset to train the model. The results obtained are 97% for validation accuracy and 98% for testing accuracy. This study applies gradient-weighted class activation mapping (Grad-CAM) to enhance model interpretability. This interpretability is vital for improving predictive performance, fostering trust, and ensuring seamless integration into agricultural practices.

## 1 Introduction

Agriculture has long relied on scientific advancements to satisfy global food needs. However, various challenges encountered by those in this industry endanger the food security of human society. Recognized risks include shifting climate patterns, the impact of livestock grazing, the spread of plant diseases, and more (Calicioglu et al., 2019). Among the numerous threats, the effects of plant diseases stand out significantly. It leads to substantial losses of crops destined for human consumption. It profoundly influences society’s health and the sustenance of farmers’ livelihoods, who primarily depend on cultivating healthy crops as their primary income source (Al-Sadi, 2017; Somowiyarjo, 2011).

Timely recognition and early detection of plant diseases facilitate the adoption of proactive strategies, effectively reducing both production losses and economic impacts. Disease identification and classification historically depended on visual evaluations by experts (Gavhale and Gawande, 2014). However, this methodology frequently proves unfeasible due to the scarcity of experts in remote places, which is an intrinsically time-consuming aspect of the procedure. Today’s advancements in Artificial Intelligence (AI), Machine Learning (ML), and Computer Vision (CV) have made it possible to develop automated approaches for detecting diseases in plant leaves (Chowdhury et al., 2021). These methods can swiftly and precisely identify such diseases without human intervention. Notably, Deep Learning (DL) has emerged as a prevalent tool within the agricultural context (Guan et al., 2023), pivotal in advancing efforts to manage, regulate, and boost agricultural output.

Deep learning, a branch of machine learning, has exhibited remarkable image analysis and pattern recognition capabilities. Convolutional Neural Networks (CNNs), a prominent class of DL models, have proven effective in image classification tasks. Numerous researchers have employed deep-learning techniques to diagnose crop diseases. For instance, Chen et al. (2017) introduced a model based on DL that accurately quantifies fruits within real-time images. Similarly, Dias et al. (2018) demonstrated the application of a CNN for semantic segmentation of apple flowers, enabling the counting of flowers on plants. Ubbens et al. (2018) also investigated using a CNN model to estimate plant leaves.

While current deep learning models designed for identifying and classifying plant leaf diseases have demonstrated impressive accuracy when applied to specific leaf image datasets, their interpretability and explainability remain an area that requires more comprehensive exploration. The level of understanding and clarity these models provide has not been extensively studied, limiting the degree of confidence in their practical adoption.

Explainable AI (XAI) is an evolving field focused on developing techniques that enhance the transparency of AI models. XAI methods aim to demystify the decision-making processes of complex models, enabling researchers, practitioners, and end-users to comprehend why a particular classification or prediction was made. In conjunction with DL algorithms, the XAI, which generates explanations comprehensible to humans for the decisions made by Artificial Intelligence (AI) systems, establishes a strong foundation for implementing imaging-based AI applications across diverse domains (van der Velden et al., 2022). Notably, this synergy has significant implications in health informatics (Bhandari et al., 2022), computer vision (Buhrmester et al., 2021), and numerous other areas.

Diverse methods, including image processing, ML, and DL, have been used to monitor and detect plant diseases, leading to substantial progress (Geetharamani and Pandian, 2019; Kamal et al., 2019; Khamparia et al., 2020). A K-means clustering segmentation technique has been employed for disease identification on potato leaves. This approach extracts features from image samples like area, color, and texture. Subsequently, algorithms based on neural networks are employed to classify and recognize diseases (Athanikar and Badar, 2016; Kumari et al., 2019). Detection of plant diseases using CNN algorithms has been favored over other deep learning algorithms (Lu et al., 2021). Jung et al. (2023) developed a deep learning model that comprises a three-step classification process to identify multiple crop diseases. This approach demonstrated a high accuracy rate of approximately 97.09% (Jung et al., 2023). ResNet50 demonstrated a 97% accuracy in identifying six common diseases of tomato leaves (Kaushik et al., 2020). Hosny et al. (2023) created a combination of lightweight deep model features and Local Binary Pattern (LBP) features to classify leaf diseases in different plants (Apple, Grape, and Tomato). This approach resulted in high accuracy (Hosny et al., 2023). Potato leaf classification for diseases was carried out using VGG16 and VGG19 architectures, resulting in an accuracy of 91% (Sholihati et al., 2020). In the work by Johnson et al. (2021), a Region Convolutional Neural Network (RCNN) model was introduced to forecast potato leaf diseases. The model’s performance was evaluated using precision and recall metrics, resulting in 98.1 and 81.9%, respectively. Mahum et al. (2023) developed a model to identify diseases on potato leaves. They harnessed the DesNet model, specifically DesNet201, enriched with an extra transition layer, to perform the classification task effectively. Employing the PlantVillage dataset, their model attained an accuracy of 97.2% (Mahum et al., 2023). Kumar and Patel (2023) suggested leveraging a hierarchy-based deep learning CNN to detect and classify potato diseases. They harnessed the intuitionist Fuzzy LBP to extract the features. The approach attained an accuracy rate reached 95.7% (Kumar and Patel, 2023). Anim-Ayeko et al. (2023) developed a ResNet-9 model to accurately identify the blight disease state in potato and tomato leaf images. The model achieved an accuracy rate of 99.25%. Additionally, the researchers explained the model’s predictions using saliency maps, which offer insights into the reasoning behind the model’s classifications (Anim-Ayeko et al., 2023). Khalifa et al. (2021) suggested a deep convolutional neural network-based architecture for classifying potato leaf blight into three categories: healthy, early, and late. The proposed model achieved a testing accuracy of 98% (Khalifa et al., 2021). Tiwari et al. (2020) utilized the pre-trained VGG-19 model to extract features from images of potato leaves, which were then categorized into three classes: early blight, late blight, and healthy. These extracted features were subsequently input into four classification models: SVM, Artificial Neural Networks, KNN, and Logistic Regression. The Logistic Regression model demonstrated the highest performance, achieving a test accuracy of 97.8% (Tiwari et al., 2020). Chakraborty et al. (2022) evaluated the performance of several contemporary deep-learning models in the automated recognition of late and early blight diseases in potato leaves using optical imagery. The researchers trained and compared four deep learning architectures, including VGG16, VGG19, MobileNet, and ResNet50, on the PlantVillage dataset. Their findings indicated that VGG16 demonstrated the highest accuracy at 92.69% compared to the other evaluated models. To further improve the VGG16 model’s performance, the researchers conducted fine-tuning by adjusting its hyperparameters. This refined methodology ultimately achieved a 97.89% accuracy in classifying late and early blight syndromes in potato leaves (Chakraborty et al., 2022).

A few researchers have envisaged combining XAI and DL models for forecasting distinct subtypes of plant leaf diseases, encompassing the incorporation of explanatory outcomes (Kinger and Kulkarni, 2021). Özbılge et al. (2022) suggested a compact CNN model for identifying tomato diseases. This model demonstrated an impressive accuracy rate of 99.55%. Furthermore, the researchers explained the model’s behavior using Grad-CAM analysis. This technique allowed them to highlight the regions within the input images most influential in CNN’s classification decisions (Özbılge et al., 2022). Masood et al. (2023) developed a deep learning-based model called MaizeNet that can accurately localize and classify various leaf disorders affecting maize crops. The approach employed the ResNet-50 architecture enhanced with spatial-channel attention mechanisms to extract discriminative features, resulting in a classification accuracy of 97.89%. Also, the authors utilized the Grad-CAM to visualize the salient image regions contributing to the network’s class predictions, providing interpretability to the model’s decision-making process (Masood et al., 2023). The research team in Quach et al. (2023) evaluated the performance of deep learning models in classifying tomato leaf images. They also emphasized the importance of using XAI techniques, such as Grad-CAM, to assess the reliability of these black-box models, particularly in the agricultural domain. The study’s findings suggested that employing XAI is crucial for developing the most trustworthy deep learning models (Quach et al., 2023). Given the notable accuracy of DL models in detecting and classifying potato leaf diseases, along with the absence of interpretability in the output of these models according to existing research works, this paper aims to construct a framework based on Deep Learning Explainable Artificial Intelligence (DL-XAI) to detect and classify the diseases of potato leaves.

This study introduces an approach for classifying potato leaf diseases involving a trained DL model. It also offers an interpretable approach that emphasizes the crucial parts of the leaves that influence disease classification. The primary aim of this DL-based XAI-guided approach is to furnish decision-making support within agriculture.

The following is a summary of the primary contributions of this paper:

1) A new DL approach is developed based on VGG16 to classify potato leaf disease.
2) A framework for classifying potato leaf diseases based on Deep Learning explainable Artificial Intelligence (DL-XAI) is developed, employing Grad-CAM to explain the outcomes of the implemented deep learning model.
3) An extensive evaluation study is conducted to gauge the dependability of the introduced DL-XAI framework.

The organization of this paper is outlined as follows: Section 2 describes the proposed model utilized in classifying potato leaf diseases. Section 3 presents the experiments, the results, and the discussion. Finally, Section 5 concludes this paper.

## 2 Materials and methods

### 2.1 Dataset

**Figure 1:**
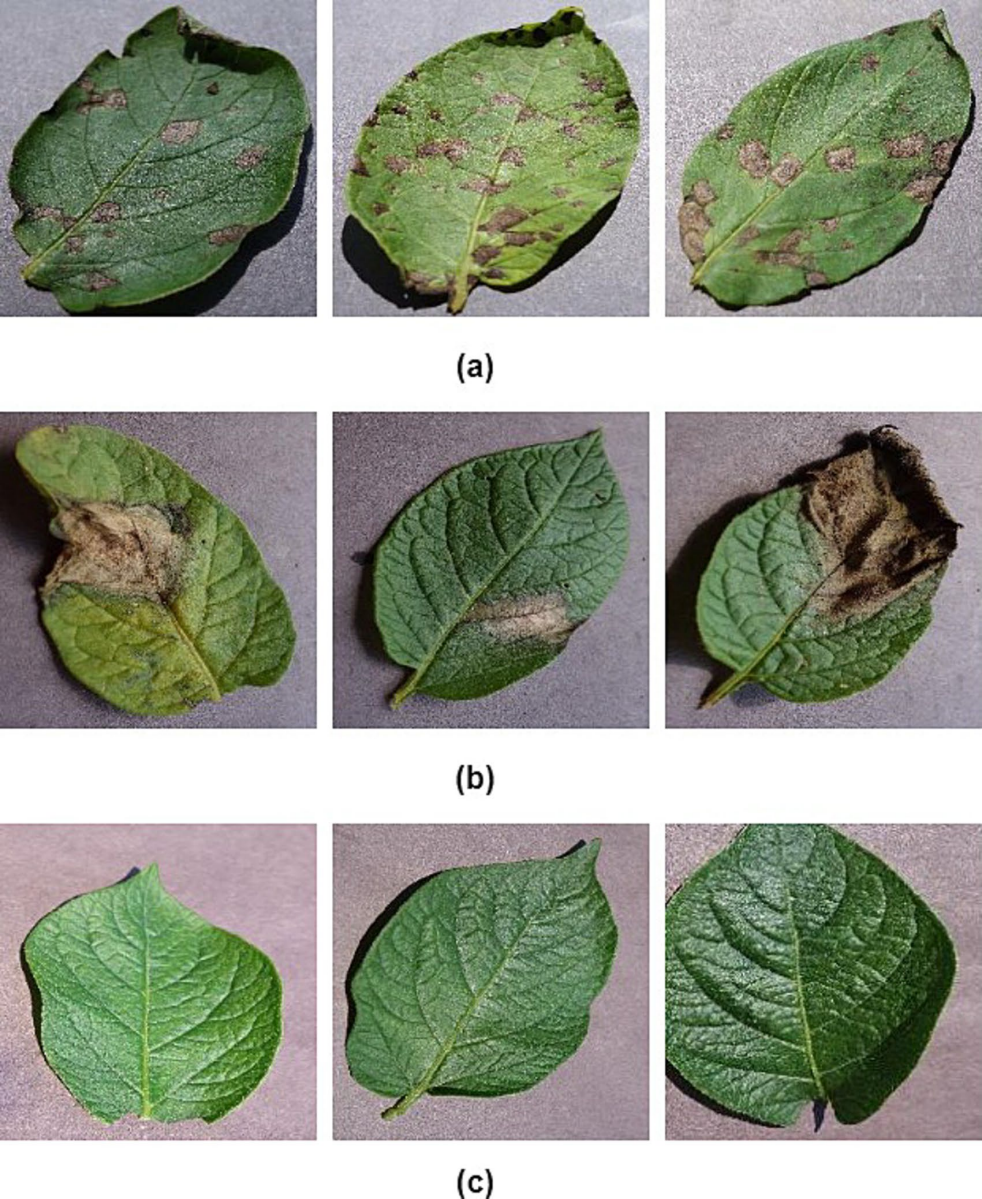
Example images of potato leaves: **(A)** early blight, **(B)** late light, and **(C)** healthy (Al-Dabbagh, 2022).

To attain the aims of this research, we employ a standardized dataset for potato leaf disease derived from the openly accessible PlantVillage dataset (Al-Dabbagh, 2022). The potato leaf dataset consists of 2,152 images. These image samples are depicted in Figure 1. The data is categorized into three distinct categories, as demonstrated in Table 1. Data augmentation techniques are applied to expand the size of the potato leaf disease dataset while retaining its intrinsic biological characteristics. Specifically, we utilize prevalent geometric transformation techniques, including rotation, scaling, flipping, and vertical translation. The dataset is partitioned, with 80% for training and 20% for testing. The training dataset is divided into training and validation sets. 15% of the training data is set aside as a validation subset to avoid over-fitting (Kamilaris and Prenafeta-Boldú, 2018).

**Table 1:** Distribution of potato leaf disease dataset for each class.

Class	Samples	
	Before using data augmentation	After using data augmentation
Early blight	1,000	1,000
Late blight	1,000	1,000
Healthy	152	1,000
Total	2,152	3,000

### 2.2 The proposed model

**Figure 2:**
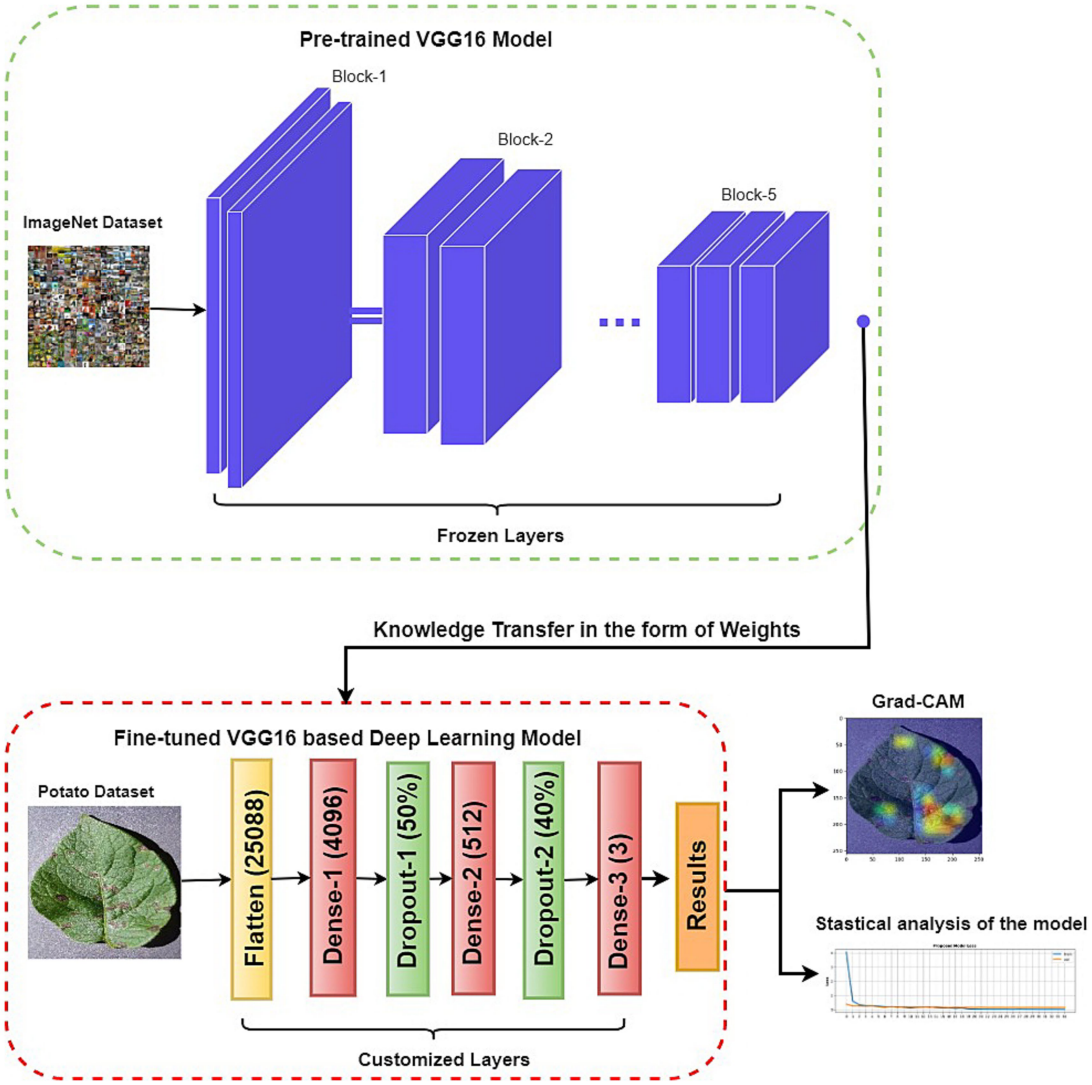
The structure of the proposed approach for potato leaf disease classification.

The proposed approach comprises two key components: a customized VGG16 architecture and XAI techniques. This hybrid architecture capitalizes on deep learning and interpretability strengths to yield accurate and understandable disease classifications. Also, the proposed XAI framework validates that the neural network has learned accurate attributes, thereby bolstering confidence in its predictions. Figure 2 illustrates the conceptual layout of the proposed model.

#### 2.2.1 VGG16 model

VGG16, a deep CNN model introduced by Simonyan and Zisserman (2014), attained an impressive top 5 test accuracy, reached 92.7% on the ImageNet dataset, and emerged as the winner in the Large-Scale Visual Recognition Challenge (ILSVRC) competition conducted by the Oxford Visual Geometry Group (Guan and Loew, 2017; Montaha et al., 2021). VGG model’s heightened depth enables kernels to grasp more intricate features. In studies assessing the efficacy of transfer learning (Mehra, 2018), it was ascertained that a fine-tuned VGG16 model that has already been trained attained notably superior accuracy compared to a completely trained network.

#### 2.2.2 Customized VGG16 model

This paper uses a framework for building and fine-tuning a VGG16-based model to classify potato leaf diseases into three distinct classes: early blight, late blight, and healthy. The dimensions of the potato leaf images are adjusted to 224 by 224 pixels.

Initially, we utilize a pre-trained VGG16 model that has gained valuable features from an extensive dataset like ImageNet. We import this VGG16 model and its pre-trained weights but omit its original classification layers, which are meant for different classes. Subsequently, the weights of the convolutional layers are frozen to retain the capabilities acquired during pre-training. Finally, we formulate the customized classification layers to be integrated with the VGG16 base mode. Additionally, dropout layers are added to prevent overfitting.

#### 2.2.3 XAI-based methods

Deep learning networks are often called “black boxes” due to their inability to offer insights into which specific input elements contributed to the network’s predictions or the nature of the knowledge it has acquired. When producing incorrect predictions, these models often experience significant failures without prior indication or clarification. Class activation mapping is a technique employed to attain visual explanations for the predictions rendered by convolutional neural networks. Erroneous predictions that may seem inexplicable possess logical justifications. We used class activation mapping (CAM) to investigate whether specific areas within an input image perplexed the network, leading to inaccurate predictions. To attain this analysis, we used the Gradient-weighted Class Activation Mapping (Grad-CAM) technique.

The XAI framework for potato leaf disease classification incorporates the Grad-CAM technique, which generates class activation maps. Grad-CAM generates weight maps that accentuate significant regions within the input image that CNN has relied upon to make its class label prediction. This method capitalizes on the gradient values propagated through the final convolutional layer to produce these informative class activation maps (Selvaraju et al., 2017). Grad-CAM mapping concerning a specific class C with N pixel is explained in Equation 1.


(1) $$M^{C}=\mathrm{ReLU}\left(\underset{k}{\sum }\alpha_{C}^{K}A^{K}\right)$$

where, $M^{C}$ is a class activation map for the target class C which highlights the spatial regions in the input image that were important for the model’s prediction of class C, $A^{K}$ , is the feature map from a specific convolutional layer of the neural network., and the gradients-based significance weights for class C and feature map K ( $\alpha_{C}^{K})$ , is computed in Equation 2 as (Selvaraju et al., 2017):


(2) $$\alpha_{C}^{K}=\frac{1}{N}\underset{i,j}{\sum }\left(\frac{dy^{C}}{dA_{i.j}^{K}}\right)$$

$\alpha_{C}^{K}$ measure how much each feature map $A^{K}$ contributes to the target class C where, $y^{C}$ is the score for class C.

## 3 Experimental results and discussion

### 3.1 Environmental execution

The experimental execution of the proposed DL-based XAI framework was accomplished utilizing the TensorFlow framework and the open-source Keras libraries. The training process employed the Adam optimizer, the categorical cross-entropy loss function, and other hyperparameters (see Table 2). These experimental procedures were carried out on the Google Colab platform, serving as the environment for implementing the DL-based XAI model.

**Table 2:** Training hyperparameter for the proposed model.

Parameter	Setting
Algorithm optimization	Adam
Learning rate	0.001
Batch size	32
Epochs	100

### 3.2 Evaluation metrics

Assessing the suggested approach’s effectiveness involves computing several performance metrics, including accuracy, precision, recall, F1-score, the area under the ROC curve (AUC-ROC), and the confusion matrix (Stojanović et al., 2014) (see Equation 3–6). These measures collectively evaluate the suggested method’s robustness and credibility.


(3) $$\mathrm{Accuracy}=\frac{t_{p}+t_{n}}{t_{p}+f_{p}+f_{n}+t_{n}}$$

(4) $$\mathrm{Precision}=\frac{t_{p}}{t_{p}+f_{p}}$$

(5) $$\mathrm{Recall}=\frac{t_{p}}{t_{p}+f_{n}}$$

(6) $$F1\;\mathrm{score}=\frac{2t_{p}}{2t_{p}+f_{p}+f_{n}}$$

Where $t_{p},t_{n},f_{p},\mathrm{and}\;f_{n}$ denote true positive, true negative, false positive, and false negative, respectively.

## 4 Results and discussion

Four advanced deep learning models, including VGG16 (Simonyan and Zisserman, 2014), InceptionResNetV2 (Szegedy et al., 2017), GoogleNet (Szegedy et al., 2016), and AlexNet (Krizhevsky et al., 2012), were examined for the backbone network. These models utilized identical training configurations and system arrangements. The classification results are detailed in Table 3, with VGG16 demonstrating superior performance in accuracy, precision, recall, and F1-score compared to the other pre-trained models. As a result, the VGG16 model was incorporated into our proposed XAI framework.

**Table 3:** Classification assessment performance of the pre-trained deep learning models.

Model	Precision	Recall	F1-score	Accuracy
AlexNet	96%	96%	96%	95.6%
GoogleNet	96%	96%	96%	96%
InceptionResNetV2	97%	97%	97%	96.6%
VGG16	98%	98%	98%	98%

### 4.1 Model explanation with customized VGG16

**Figure 3:**
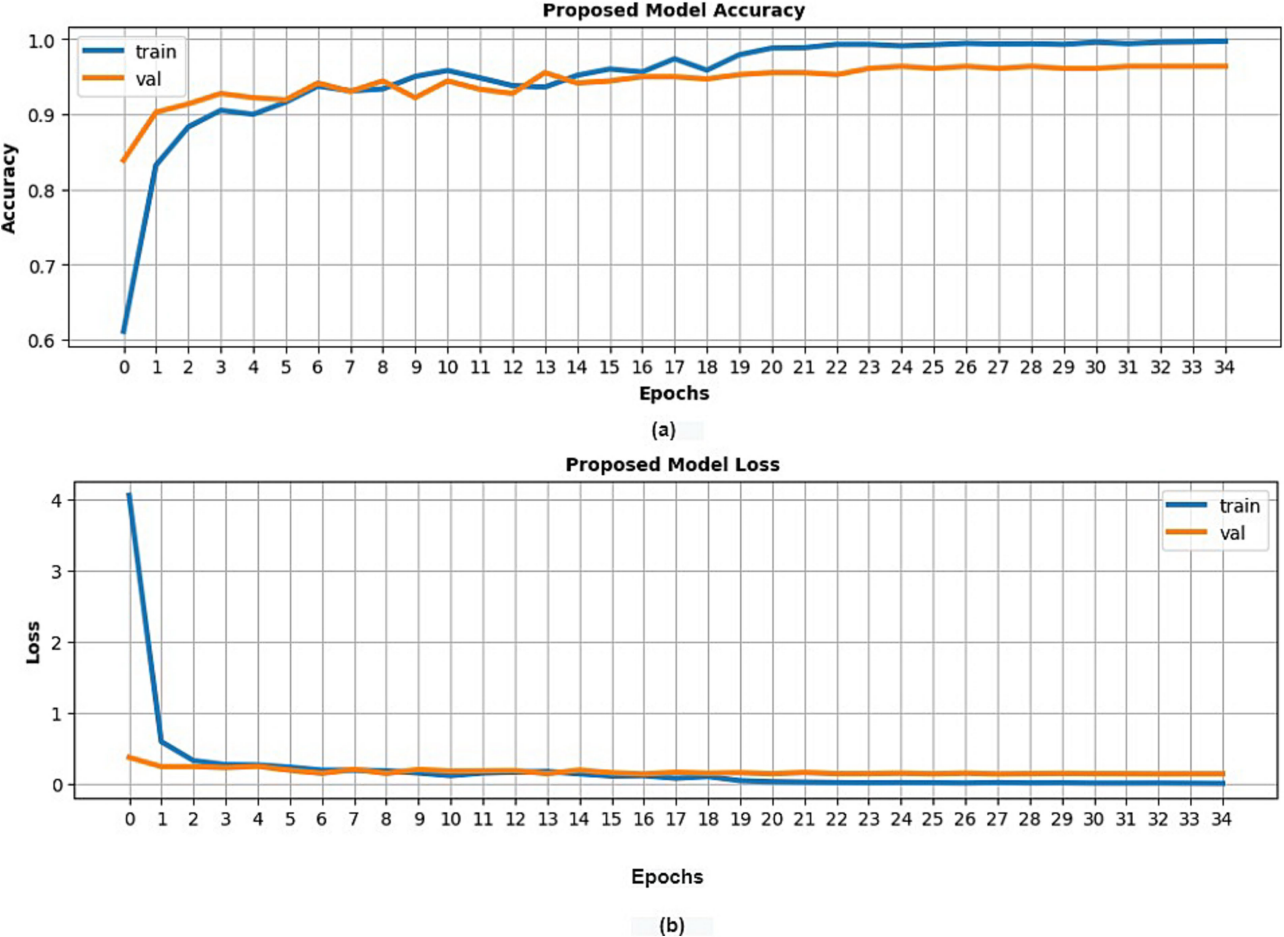
Classification results of the presented model (customized VGG16) with potato leaf dataset **(A)** training and validation accuracy **(B)** training and validation loss.

Conventional statistical validation procedures were applied, which involved evaluating model performance using metrics such as loss and accuracy across training, validation, and test datasets. Additionally, precision, recall, and F1-score were computed. A predefined stopping criterion of 50 epochs was set for the model’s training. As depicted in Figure 3, the suggested model attained a training accuracy rate of 99.75% and a validation accuracy of 97%. The model’s performance was further evaluated on a previously unseen test set that wasn’t used during the training phase. This evaluation resulted in a test accuracy of 98%. Also, the precision, recall, and F1 scores of each category are depicted in Table 4.

**Table 4:** Precision, recall, and F1-core for each potato leaf disease class on the test dataset.

Model	Precision	Recall	F1-Score
Early blight	0.99	0.98	0.99
Late blight	0.96	0.98	0.97
Healthy	0.99	0.97	0.98
Accuracy			0.98
Macro_Avg	0.98	0.98	0.98
Weighted_Avg	0.98	0.98	0.98

**Figure 4:**
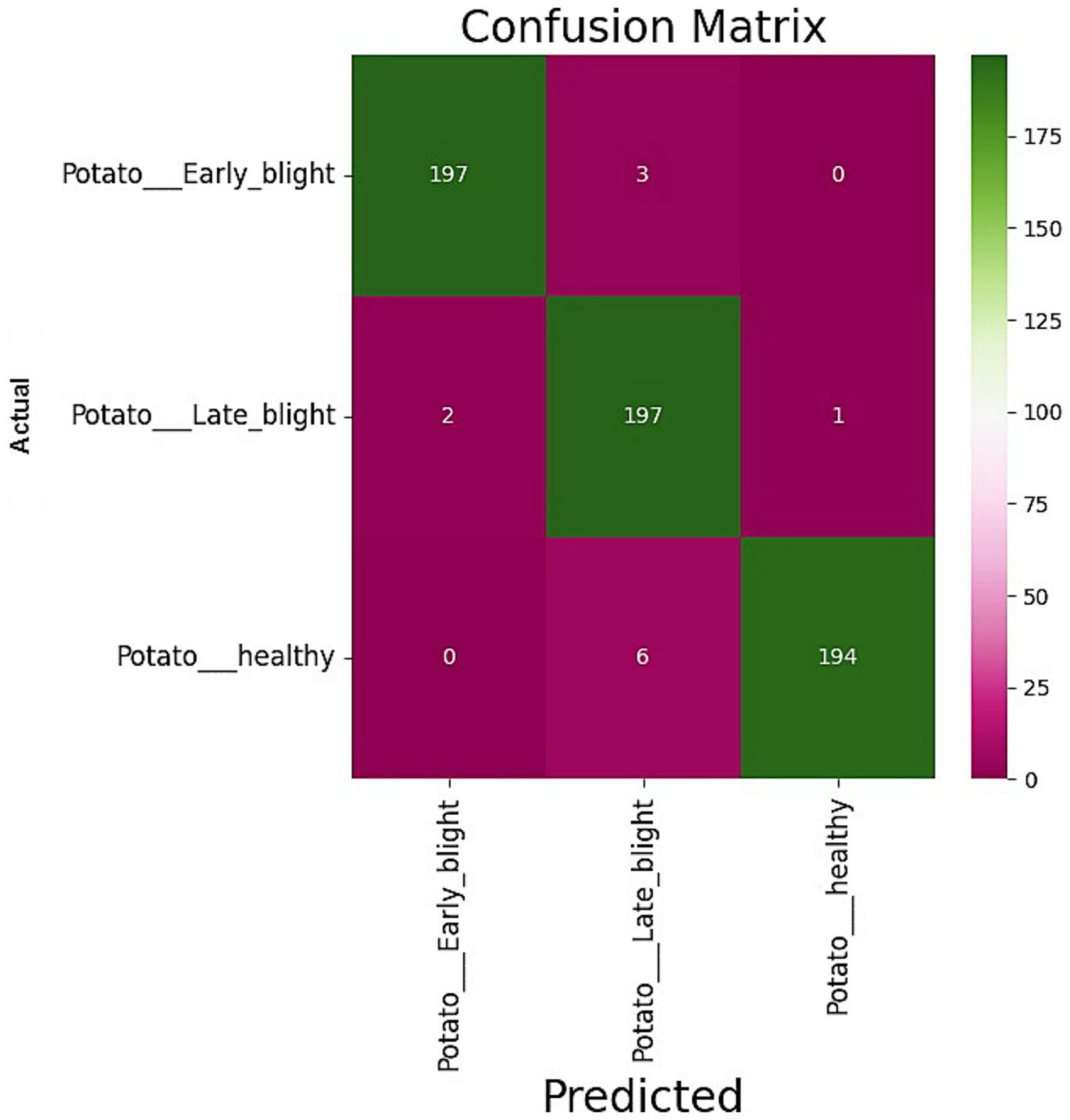
Confusion matrix obtained by the proposed model.

**Figure 5:**
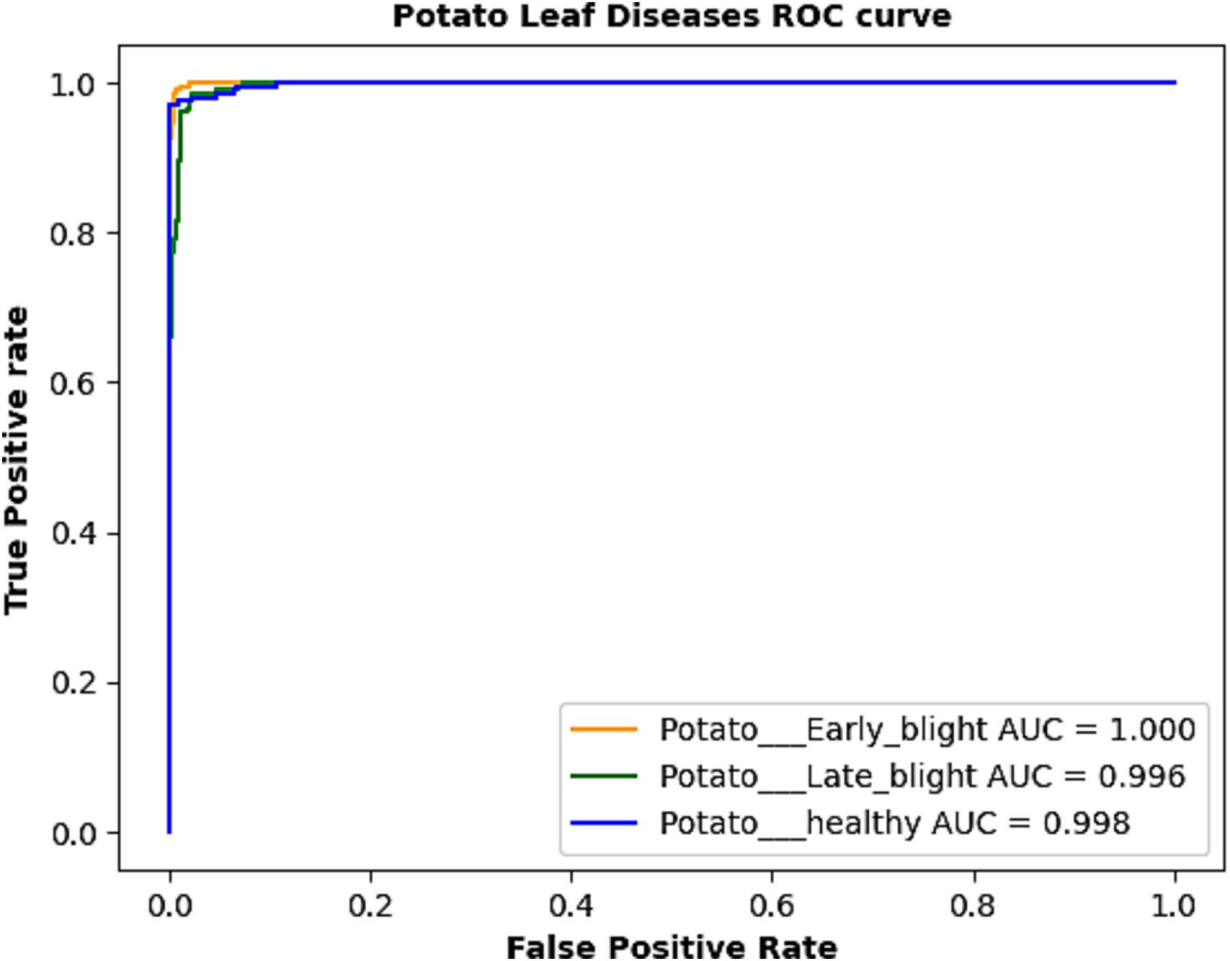
AUC-ROC curve obtained by the proposed model.

The confusion matrix of the suggested model is depicted in Figure 4. We assessed the AUC- ROC scores for each class to evaluate the efficacy of the proposed model, as illustrated in Figure 5. The customized VGG16 model designed for potato leaf disease classification exhibited effective performance across all classes, as indicated by the AUC-ROC values specific to each class (Figure 5). These results highlight the model’s efficacy in effectively addressing the challenges of multi-class categorization. The high classification accuracy of the proposed DL model for potato leaf diseases can help farmers more effectively manage their crop growing. Accurate and reliable disease identification enables farmers to make more informed decisions regarding the timing and application of treatments, potentially mitigating crop losses and enhancing yield quality. By promptly detecting and classifying diseases, farmers can optimize agricultural inputs like pesticides and fertilizers, applying them judiciously. This precision-based approach can yield cost savings, diminish environmental impacts, and promote more sustainable farming practices.

### 4.2 Model explanation with XAI

The Grad-CAM technique was utilized to identify significant regions within potato leaf disease images that played a crucial role in the classification process. This goal was achieved by harnessing the spatial information preserved within the convolutional layers. A thorough analysis of individual potato leaf disease samples from each category was undertaken to assess the effectiveness of the proposed visual explanation techniques. This analysis included visually inspecting the heatmaps produced by the methodologies. Table 5 presents the resulting heatmap. While Grad-CAM visually represents salient features, the resulting heatmaps can be equivocal. The efficacy of Grad-CAM depends on the DL model architecture. In highly complex or deep models, the heatmaps may be more challenging to interpret as the model’s internal decision-making processes become more abstract, which can impede the provision of clear and actionable explanations.

**Table 5:** XAI framework result for the proposed model.

Class	Potato leaf	Grad-CAM
Early_blight	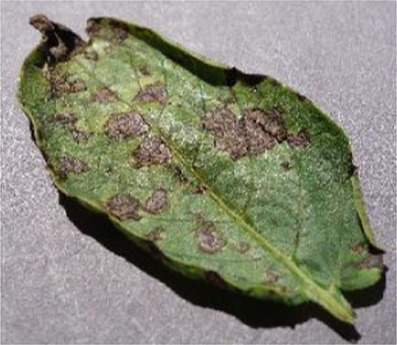	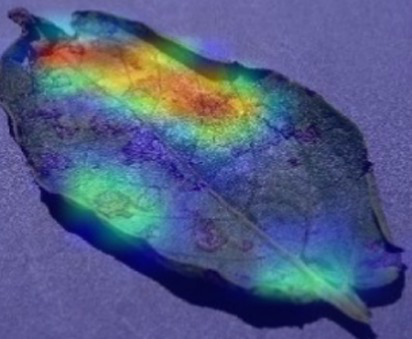
Late_blight	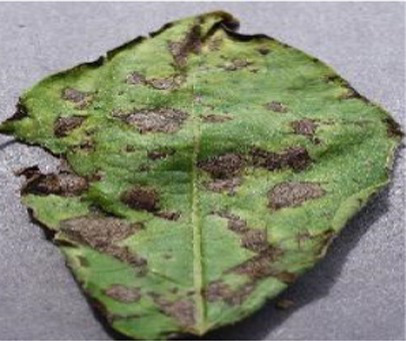	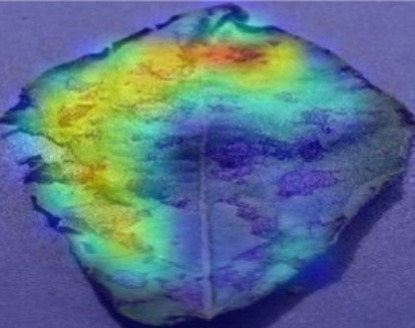
Healthy	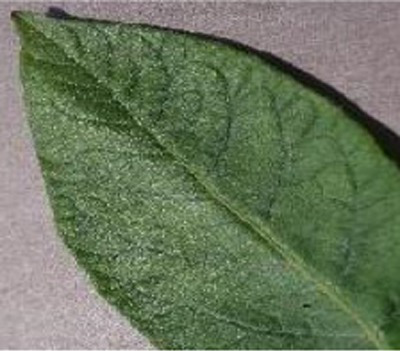	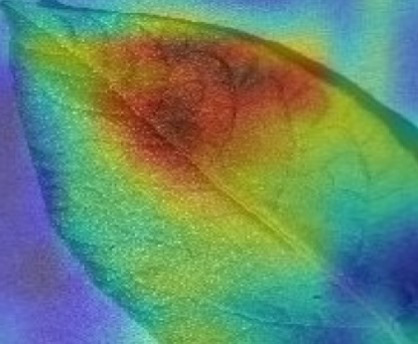

### 4.3 Comparison with the existing approaches

Table 6 compares the classification performance between the presented model and previously established methods for potato leaf disease. The presented model demonstrates superior performance compared to all the existing approaches in the table, showcasing a notable enhancement in accuracy. Notably, our model is the only one on the table that employs XAI.

**Table 6:** Comparison with the existing methods for potato leaf disease.

Authors	Method	Accuracy (%)	XAI
Kurmi et al. (2022)	CNN	95.35	No
Mahum et al. (2023)	Efficient DenseNet	97.2	No
Khalifa et al. (2021)	DCNN	98	No
Sanjeev et al. (2021)	ANN	96.5	No
Proposed model	Customized VGG16	98	Grad-CAM

## 5 Conclusion

In this research, we proposed an XAI framework and a transfer learning-based DL model for potato leaf disease classification—the architecture of our model leveraged transfer learning with the pre-trained VGG16 model to furnish a solution. Insightful explanations drive this solution. Additionally, the Grad_CAM method generated a detailed explanation of the presented model results. The validation and test accuracies achieved by the proposed model were 97 and 98%, respectively. The utilization of Grad-CAM for explanation generation successfully pinpointed the precise regions responsible for the categorization of potato leaf disease. The introduced model underscores the efficacy of combining XAI techniques with a tailored VGG16 architecture, as it produced admissible explanations for the outcomes while maintaining a high classification accuracy.

In the future, we suggest exploring and utilizing additional methods for XAI (Paccotacya-Yanque et al., 2024), including LIME, SHAP, Grad-CAM++, and HiResCAM, to obtain more detailed and comprehensive explanations. These additional methods can enhance the model’s understanding and help it make better-informed decisions. Additionally, Expanding the dataset to include images representing greater geographic, temporal, and cultivar diversity could enhance the model’s robustness and generalization ability. Also, Incorporating the model with real-time data acquisition tools, like IoT sensor networks, could enhance its reactivity and precision in practical settings, enabling farmers to receive prompt and applicable information.

## Data Availability

Publicly available datasets were analyzed in this study. This data can be found at: Kaggle.

## References

[ref1] Al-Dabbagh A. (2022). PlantVillage Dataset. Available at: https://www.kaggle.com/datasets/abdallahalidev/plantvillage-dataset (Accessed August 20, 2022).

[ref2] Al-Sadi A. M. (2017). Impact of plant diseases on human health. Int. J. Nutr. Pharmacol. Neurol. Dis. 7, 21–22. doi: 10.4103/ijnpnd.ijnpnd_24_17

[ref3] Anim-Ayeko A. O. Schillaci C. Lipani A. (2023). Automatic blight disease detection in potato (*Solanum tuberosum* L.) and tomato (*Solanum lycopersicum*, L. 1753) plants using deep learning. Smart Agric. Technol. 4:100178. doi: 10.1016/j.atech.2023.100178, PMID: 39743835

[ref4] Athanikar G. Badar P. (2016). Potato leaf diseases detection and classification system. Int. J. Comput. Sci. Mobile Comput. 5, 76–88. doi: 10.1109/ICICCS48265.2020.9121067

[ref5] Bhandari M. Shahi T. B. Siku B. Neupane A. (2022). Explanatory classification of CXR images into COVID-19, pneumonia, and tuberculosis using deep learning and XAI. Comput. Biol. Med. 150:106156. doi: 10.1016/j.compbiomed.2022.106156, PMID: 36228463 PMC9549800

[ref6] Buhrmester V. Münch D. Arens M. (2021). Analysis of explainers of black box deep neural networks for computer vision: a survey. Mach. Learn. Knowl. Extr. 3, 966–989. doi: 10.3390/make3040048

[ref7] Calicioglu O. Flammini A. Bracco S. Bellù L. Sims R. (2019). The future challenges of food and agriculture: an integrated analysis of trends and solutions. Sustain. For. 11:222. doi: 10.3390/su11010222

[ref8] Chakraborty K. K. Mukherjee R. Chakraborty C. Bora K. (2022). Automated recognition of optical image-based potato leaf blight diseases using deep learning. Physiol. Mol. Plant Pathol. 117:101781. doi: 10.1016/j.pmpp.2021.101781

[ref9] Chen S. W. Shivakumar S. S. Dcunha S. das J. Okon E. Qu C.. (2017). Counting apples and oranges with deep learning: a data-driven approach. IEEE Robot. Autom. Lett. 2, 781–788. doi: 10.1109/LRA.2017.2651944

[ref10] Chowdhury M. E. H. Rahman T. Khandakar A. Ayari M. A. Khan A. U. Khan M. S.. (2021). Automatic and reliable leaf disease detection using deep learning techniques. AgriEngineering 3, 294–312. doi: 10.3390/agriengineering3020020

[ref11] Dias P. A. Tabb A. Medeiros H. (2018). Multispecies fruit flower detection using a refined semantic segmentation network. IEEE Robot. Autom. Lett. 3, 3003–3010. doi: 10.1109/LRA.2018.2849498

[ref12] Gavhale K. R. Gawande U. (2014). An overview of the research on plant leaves disease detection using image processing techniques. IOSR J. Comput. Eng. 16, 10–16. doi: 10.9790/0661-16151016

[ref13] Geetharamani G. Pandian A. (2019). Identification of plant leaf diseases using a nine-layer deep convolutional neural network. Comput. Electr. Eng. 76, 323–338. doi: 10.1016/j.compeleceng.2019.04.011, PMID: 39743835

[ref14] Guan H. Fu C. Zhang G. Li K. Wang P. Zhu Z. (2023). A lightweight model for efficient identification of plant diseases and pests based on deep learning. Front. Plant Sci. 14:1227011. doi: 10.3389/fpls.2023.1227011, PMID: 37521914 PMC10382237

[ref15] Guan S. Loew M. (2017) "Breast cancer detection using transfer learning in convolutional neural networks," *in 2017 IEEE Appl. Imagery Pattern Recognit. Workshop (AIPR)*, pp. 1–8, October 2017.

[ref16] Hosny K. M. El-Hady W. M. Samy F. M. Vrochidou E. Papakostas G. A. (2023). Multi-class classification of plant leaf diseases using feature fusion of deep convolutional neural network and local binary pattern. IEEE Access 11, 62307–62317. doi: 10.1109/ACCESS.2023.3286730

[ref17] Johnson J. Sharma G. Srinivasan S. Masakapalli S. K. Sharma S. Sharma J.. (2021). Enhanced field-based detection of potato blight in complex backgrounds using deep learning. Plant Phenomics, 2021. doi: 10.34133/2021/9835724, PMID: 34104897 PMC8147694

[ref18] Jung M. Song J. S. Shin A. Y. Choi B. Go S. Kwon S. Y.. (2023). Construction of deep learning-based disease detection model in plants. Sci. Rep. 13:7331. doi: 10.1038/s41598-023-34549-2, PMID: 37147432 PMC10163233

[ref19] Kamal K. C. Yin Z. Wu M. Wu Z. (2019). Depthwise separable convolution architectures for plant disease classification. Comput. Electron. Agric. 165:104948. doi: 10.1016/j.compag.2019.104948

[ref20] Kamilaris A. Prenafeta-Boldú F. X. (2018). Deep learning in agriculture: a survey. Comput. Electron. Agric. 147, 70–90. doi: 10.1016/j.compag.2018.02.016

[ref21] Kaushik M. Prakash P. Ajay R. Veni S. (2020), "Tomato leaf disease detection using convolutional neural network with data augmentation," *in 2020 5th Int. Conf. Commun. Electron. Syst. (ICCES)*, pp. 1125–1132, June 2020.

[ref22] Khalifa N. E. M. Taha M. H. N. Abou El-Maged L. M. Hassanien A. E. (2021) "Artificial intelligence in potato leaf disease classification: a deep learning approach," *in Machine Learning and BigData Analytics Paradigms: Analysis, Applications and Challenges,* 2021, pp. 63–79.

[ref23] Khamparia A. Saini G. Gupta D. Khanna A. Tiwari S. de Albuquerque V. H. C. (2020). Seasonal crops disease prediction and classification using deep convolutional encoder network. Circuits Syst. Signal Process. 39, 818–836. doi: 10.1007/s00034-019-01041-0

[ref24] Kinger S. Kulkarni V. (2021) "Explainable A.I. For deep learning-based disease detection," *in 2021 Thirteenth Int. Conf. Contemp. Comput. (IC3-2021)*, pp. 209–216, August 2021.

[ref25] Krizhevsky A. Sutskever I. Hinton G. E. (2012) "Imagenet classification with deep convolutional neural networks," *in Advances in neural information processing systems*, vol. 25.

[ref26] Kumar A. Patel V. K. (2023). Classification and identification of disease in potato leaf using hierarchical-based deep learning convolutional neural network. Multimed. Tools Appl. 82, 31101–31127. doi: 10.1007/s11042-023-14663-z, PMID: 39744507

[ref27] Kumari C. U. Prasad S. J. Mounika G. (2019), "Leaf disease detection: feature extraction with K-means clustering and classification with ANN," *in 2019 3rd Int. Conf. Comput. Methodologies Commun. (ICCMC)*, pp. 1095–1098, March 2019.

[ref28] Kurmi Y. Saxena P. Kirar B. S. Gangwar S. Chaurasia V. Goel A. (2022). Deep CNN model for crops' diseases detection using leaf images. Multidimens. Syst. Signal Process. 33, 981–1000. doi: 10.1007/s11045-022-00820-4

[ref29] Lu J. Tan L. Jiang H. (2021). Review on convolutional neural network (CNN) applied to plant leaf disease classification. Agriculture 11:707. doi: 10.3390/agriculture11080707

[ref30] Mahum R. Munir H. Mughal Z. U. N. Awais M. Sher Khan F. Saqlain M.. (2023). A novel framework for potato leaf disease detection using an efficient deep learning model. Hum. Ecol. Risk Assess. Int. J. 29, 303–326. doi: 10.1080/10807039.2022.2064814

[ref31] Masood M. Nawaz M. Nazir T. Javed A. Alkanhel R. Elmannai H.. (2023). MaizeNet: a deep learning approach for effective recognition of maize plant leaf diseases. IEEE Access 11, 52862–52876. doi: 10.1109/ACCESS.2023.3280260

[ref32] Mehra R. (2018). Breast cancer histology images classification: training from scratch or transfer learning? ICT Express 4, 247–254. doi: 10.1016/j.icte.2018.10.007, PMID: 39743835

[ref33] Montaha S. Azam S. Rafid A. K. M. R. H. Ghosh P. Hasan M. Z. Jonkman M.. (2021). BreastNet18: a high-accuracy fine-tuned VGG16 model evaluated using ablation study for diagnosing breast cancer from enhanced mammography images. Biology 10:1347. doi: 10.3390/biology10121347, PMID: 34943262 PMC8698892

[ref34] Özbılge E. Ulukök M. K. Toygar Ö. Ozbılge E. (2022). Tomato disease recognition using a compact convolutional neural network. IEEE Access 10, 77213–77224. doi: 10.1109/ACCESS.2022.3192428

[ref35] Paccotacya-Yanque R. Y. Bissoto A. Avila S. (2024), "Are explanations helpful? A comparative analysis of explainability methods in skin lesion classifiers," in 2024 20th International Symposium on Medical Information Processing and Analysis (SIPAIM), 1–5, November 2024. doi: 10.1109/SIPAIM62974.2024.10783606

[ref36] Quach L. D. Nguyen K. Q. Nguyen A. Q. Thai-Nghe N. Nguyen T. G. (2023). Explainable deep learning models with gradient-weighted class activation mapping for smart agriculture. IEEE Access 11, 83752–83762. doi: 10.1109/ACCESS.2023.3296792, PMID: 39573497

[ref37] Sanjeev K. Gupta N. K. Jeberson W. Paswan S. (2021). Early prediction of potato leaf diseases using ANN classifier. Oriental J. Comput. Sci. Technol 13, 129–134. doi: 10.13005/ojcst13.0203.11

[ref38] Selvaraju R. R. Cogswell M. Das A. Vedantam R. Parikh D. Batra D. (2017), "Grad-CAM: visual explanations from deep networks via gradient-based localization," *in Proceedings of the IEEE International Conference on Computer Vision*, 2017, pp. 618–626.

[ref39] Sholihati R. A. Sulistijono I. A. Risnumawan A. Kusumawati E. (2020) "Potato leaf disease classification using deep learning approach," *in 2020 Int. Electron. Symp. (IES)*, pp. 392–397.

[ref40] Simonyan K. Zisserman A. (2014). "Very deep convolutional networks for large-scale image recognition," *arXiv preprint arXiv:1409.1556*

[ref41] Somowiyarjo S. (2011). Plant disease problems on smallholder farms in Asia. Australas. Plant Pathol. 40, 318–319. doi: 10.1007/s13313-011-0075-5

[ref42] Stojanović M. Apostolović M. Stojanović D. Milošević Z. Toplaović A. Mitić-Lakušić V.. (2014). Understanding sensitivity, specificity, and predictive values. Vojnosanit. Pregl. 71, 1062–1065. doi: 10.2298/VSP1411062S 25536811

[ref43] Szegedy C. Ioffe S. Vanhoucke V. Alemi A. (2017). "Inception-v4, inception-Resnet, and the impact of residual connections on learning," *in Proceedings of the AAAI Conference on Artificial Intelligence*, vol. 31, no. 1.

[ref44] Szegedy C. Liu W. Jia Y. Sermanet P. Reed S. Anguelov D.. (2016). Going deeper with convolutions (GoogleLeNet). J. Chem. Technol. Biotechnol. 1–9. doi: 10.1109/CVPR.2015.7298594

[ref45] Tiwari D. Ashish M. Gangwar N. Sharma A. Patel S. Bhardwaj S. (2020). Potato leaf diseases detection using deep learning. In *2020 4th international conference on intelligent computing and control systems (ICICCS)* (pp. 461–466). IEEE.

[ref46] Ubbens J. Cieslak M. Prusinkiewicz P. Stavness I. (2018). The use of plant models in deep learning: an application to leaf counting in rosette plants. Plant Methods 14, 1–10. doi: 10.1186/s13007-018-0273-z, PMID: 29375647 PMC5773030

[ref47] van der Velden B. H. Kuijf H. J. Gilhuijs K. G. Viergever M. A. (2022). Explainable artificial intelligence (XAI) in deep learning-based medical image analysis. Med. Image Anal. 79:102470. doi: 10.1016/j.media.2022.102470, PMID: 35576821

